# Ultrafast polarization control by terahertz fields via π-electron wavefunction changes in hydrogen-bonded molecular ferroelectrics

**DOI:** 10.1038/s41598-018-33076-9

**Published:** 2018-10-09

**Authors:** T. Miyamoto, D. Hata, T. Morimoto, H. Yamakawa, N. Kida, T. Terashige, K. Iwano, H. Kishida, S. Horiuchi, H. Okamoto

**Affiliations:** 1 Department of Advanced Materials Science, University of Tokyo, Kashiwa, 277-8561 Japan; 2 AIST-UTokyo Advanced Operando-Measurement Technology Open Innovation Laboratory (OPERANDO-OIL), National Institute of Advanced Industrial Science and Technology (AIST), Chiba, 277-8568 Japan; 3 Graduate University for Advanced Studies, Institute of Materials Structure Science High Energy Accelerator Research Organization (KEK), Tsukuba, 305-0801 Japan; 4 Department of Applied Physics, Nagoya University, Nagoya, 464-8603 Japan; 5 National Institute of Advanced Industrial Science and Technology (AIST), Tsukuba, 305-8562 Japan

## Abstract

Rapid polarization control by an electric field in ferroelectrics is important to realize high-frequency modulation of light, which has potential applications in optical communications. To achieve this, a key strategy is to use an electronic part of ferroelectric polarization. A hydrogen-bonded molecular ferroelectric, croconic acid, is a good candidate, since π-electron polarization within each molecule is theoretically predicted to play a significant role in the ferroelectric-state formation, as well as the proton displacements. Here, we show that a sub-picosecond polarization modulation is possible in croconic acid using a terahertz pulse. The terahertz-pulse-pump second-harmonic-generation-probe and optical-reflectivity-probe spectroscopy reveal that the amplitude of polarization modulation reaches 10% via the electric-field-induced modifications of π-electron wavefunctions. Moreover, the measurement of electric-field-induced changes in the infrared molecular vibrational spectrum elucidates that the contribution of proton displacements to the polarization modulation is negligibly small. These results demonstrate the electronic nature of polarization in hydrogen-bonded molecular ferroelectrics. The ultrafast polarization control via π-electron systems observed in croconic acid is expected to be possible in many other hydrogen-bonded molecular ferroelectrics and utilized for future high-speed optical-modulation devices.

## Introduction

**Figure 1:**
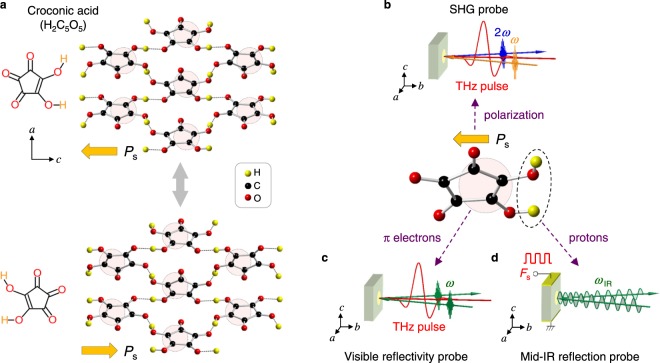
Crystal structure of croconic acid and optical probes on its polarization, π-electrons, and protons. (**a**) Molecular and crystal structure of croconic acid. The pink-shaded circles show unbalanced π-electron distributions, which are responsible for the ferroelectric polarization. (**b**–**d**) Optical probes used in this study: (**b**) terahertz-pulse-pump SHG-probe measurement for the detection of the polarization change, (**c**) terahertz-pulse-pump visible-reflectivity-probe spectroscopy for the detection of the π-electron wavefunction change, and (**d**) mid-IR reflection spectroscopy under static electric fields for the detection of the proton displacements.

Recently, several organic molecular crystals with inter-molecular hydrogen-bonds have been found to show good ferroelectricity above room temperature^1–6^. The croconic acid (H_2_C_5_O_5_) crystal shown in Fig. 1a is a representative of such ferroelectrics^2,3,6^. In this crystal, each molecule is connected with the neighbouring four molecules by hydrogen-bonds^2,7^. This crystal belongs to *Pca*2_1_ without inversion symmetry, with the ferroelectric polarization along the *c* axis. The application of an electric field above the coercive field ${F}_{{\rm{c}}}\sim 14$ kV/cm along the *c* axis induces simultaneous proton transfer and the switching of π-bond topology to reverse the polarization (Fig. 1a). It shows a large spontaneous polarization *P*_s_ of 30 μC/cm^2^ at room temperature, which is larger than *P*_s_ ~ 25 μC/cm^2^ in a famous inorganic ferroelectric, BaTiO_3_ (ref.^8^). As compared to inorganic ferroelectrics, organic molecular ferroelectrics have several advantages such as environmentally benign characteristics, low cost, and flexibility^9,10^. Another important characteristic is their ferroelectric property; theoretical studies predict that their ferroelectric polarizations are generated not only by the displacements of protons that form inter-molecular hydrogen-bonds, but also by non-centrosymmetric π-electron wavefunctions on each molecule^6^. Therefore, the ferroelectricity of hydrogen-bonded molecular ferroelectrics may not be of a simple proton-transfer type, but include a nature of “electronic ferroelectricity”^11–13^. In this sense, their ferroelectricity is different from that of typical inorganic ferroelectrics, in which the polarization originates from the sum of atomic displacements^14,15^.

Considering the unique ferroelectricity dominated by π-electron and proton dynamics in hydrogen-bonded organic molecular ferroelectrics, we expect that their macroscopic polarizations would be rapidly controlled by an external electric field^16–18^, and that this feature might have useful applications in high-frequency optical-modulation devices. In this study, we examine such a possibility by applying terahertz and static electric fields to a croconic acid crystal and measured the changes of the second-harmonic generation(SHG) (Fig. 1b), and the optical reflectivity spectra associated with π-π^*^ transitions in the visible region (Fig. 1c) and with proton vibrations in the mid-infrared(IR) region (Fig. 1d). These three kinds of measurements (Fig. 1b–d) can probe the electric-field-induced changes in macroscopic polarization, π-electron wavefunction, and proton displacements. The results reveal that the polarization is rapidly modulated by a terahertz electric field and the amplitude of polarization modulation reaches 10% at the electric field of ~150 kV/cm. The spectral changes of π-π^*^ transitions by electric fields and their theoretical simulations demonstrate that the polarization modulation originates from the electric-field-induced modifications of π-electron wavefunctions. The frequency shifts of proton vibrations by electric fields also reveal that the contribution of proton displacements to the polarization modulation is negligibly small. These results demonstrate the electronic nature of ferroelectricity in hydrogen-bonded molecular ferroelectrics.

## Results

### Terahertz-pulse-pump SHG-probe measurements to detect electric-field-induced polarization modulation

First, we discuss the results of terahertz-pulse-pump SHG-probe measurements (Fig. 1b), which detect the dynamics of polarization changes by an electric-field pulse^16–20^. Before the measurements, a static electric field *F*_s_ of ±30 kV/cm (|*F*_s_| > *F*_c_) is applied along the *c* axis to align the polarization and then it is released. When a probe pulse (0.95 eV) which is polarized parallel to the *c* axis (//*c*) is incident on the sample, a strong red SH signal (1.9 eV) appears which is visible to the unaided eyes. We ascertained that the SH light is also polarized//*c*, which is consistent with the space group (*Pca*2_1_) of the crystal.

**Figure 2:**
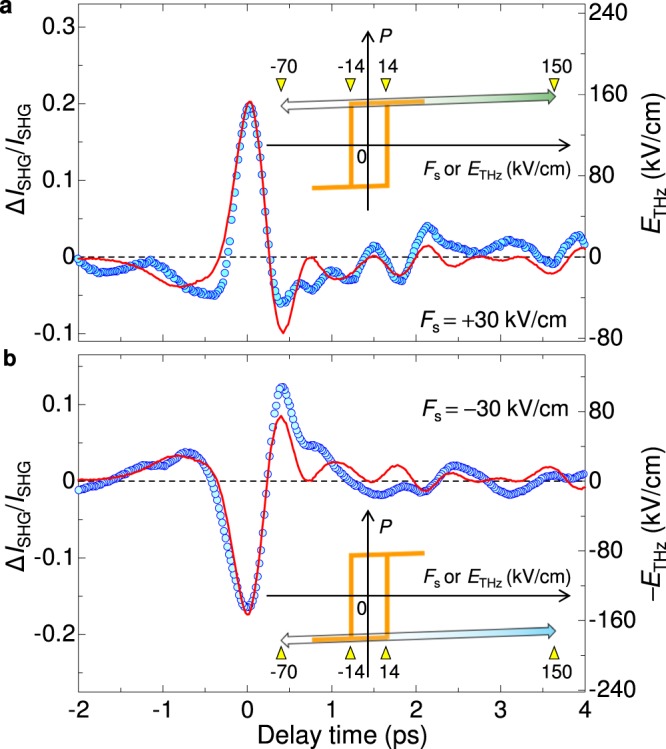
Results of terahertz-pulse-pump SHG-probe measurements. (**a**,**b**) Time evolutions of SHG-intensity changes induced by a terahertz electric-field pulse (red lines) after the static electric field, (**a**) *F*_s_ = 30 kV/cm or (**b**) −30 kV/cm, is applied. Insets show schematic *P*-*E* curves (orange lines) and polarization modulations by terahertz electric fields (arrows). The numbers in the insets show the magnitude of the electric field (kV/cm).

Figure 2a (circles) shows the time evolution of the SHG intensity $({I}_{{\rm{SHG}}})$ changes, ${\rm{\Delta }}{I}_{{\rm{SHG}}}(t)/{I}_{{\rm{SHG}}}$, by a terahertz electric-field pulse polarized//*c* after ${F}_{{\rm{s}}}=30$ kV/cm is applied. The maximum electric field |${E}_{{\rm{THz}}}(0)$| of the terahertz pulse is ~150 kV/cm. The time evolution of ${\rm{\Delta }}{I}_{{\rm{SHG}}}(t)/{I}_{{\rm{SHG}}}$ is in close agreement with a terahertz electric-field waveform, ${E}_{{\rm{THz}}}(t)$ (the red line). It is also ascertained that ${\rm{\Delta }}{I}_{{\rm{SHG}}}(0)/{I}_{{\rm{SHG}}}$ is proportional to ${E}_{{\rm{THz}}}(0)$ (see Supplementary Note 1). |${\rm{\Delta }}{I}_{{\rm{SHG}}}(0)/{I}_{{\rm{SHG}}}$| is very large, reaching ~20%. Next, we inverted the direction of the ferroelectric polarization of croconic acid by applying the reverse static electric field ${F}_{{\rm{s}}}=-\,30$ kV/cm. After that we measured ${\rm{\Delta }}{I}_{{\rm{SHG}}}(t)/{I}_{{\rm{SHG}}}$ by applying the same terahertz electric field. In this case, the sign of ${\rm{\Delta }}{I}_{{\rm{SHG}}}(t)/{I}_{{\rm{SHG}}}$ is inverted and ${\rm{\Delta }}{I}_{{\rm{SHG}}}(t)/{I}_{{\rm{SHG}}}$ almost follows −${E}_{{\rm{THz}}}(t)$ shown by the red line in Fig. 2b. Thus, the observed sub-picosecond responses are proportional to ${E}_{{\rm{THz}}}(t)$. This indicates that the polarization amplitude is modulated along the upper or lower line of the *P*-*E* curve, as illustrated in the insets of Fig. 2a,b. The polarization modulation is attributable to the sum of the modulations of dipole moment in each molecule, the details of which are discussed in the following subsections. Although |${E}_{{\rm{THz}}}(0)$| (~150 kV/cm) far exceeds *F*_c_ (~14 kV/cm), no polarization reversals are observed. This is because domain wall motions occur in the time scale of milliseconds^2,6^, and are much slower than the sub-picosecond electric-field changes within the terahertz pulse. It is natural to consider that the ultrafast responses of ${\rm{\Delta }}{I}_{{\rm{SHG}}}(t)/{I}_{{\rm{SHG}}}$ are related to modulations of *π*-electron wavefunctions or proton displacements by the terahertz electric field.

### Terahertz-pulse-pump optical-reflectivity-probe spectroscopy to detect electric-field-induced changes in π-π^*^ transitions

**Figure 3:**
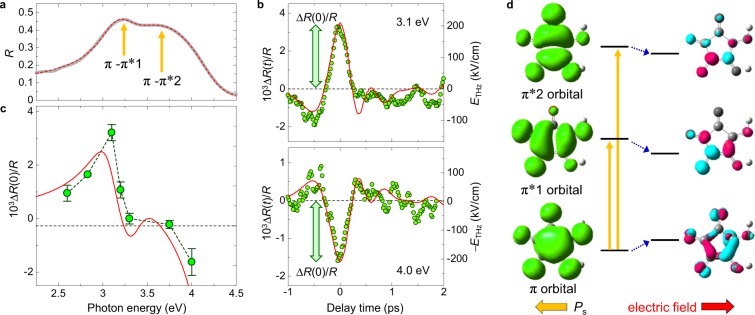
Terahertz-pulse-pump optical-reflectivity-probe spectroscopy and calculated EDDs in π-molecular orbitals. (**a**) Polarized reflectivity(*R*) spectrum for *E*//*c* (the gray line) and the fitting curve (the red broken line). (**b**) Time evolutions of reflectivity changes ${\rm{\Delta }}R(t)$/$R$ at 3.1 eV and 4.0 eV (green circles) by the terahertz electric-field ${E}_{{\rm{THz}}}(t)$ (red lines). (**c**) ${\rm{\Delta }}R(0)$/$R$ spectrum at ${E}_{{\rm{THz}}}(0)=210$ kV/cm (green circles). The red line shows the simulation curve (see text). (**d**) Calculated EDDs in π-molecular orbitals in the ground state and the excited states, π^*^1 and π^*^2 (the left part), and changes of EDDs by a static electric field *E*_s_ = 5 MV/cm opposite to the spontaneous polarization (*E*_s_//−*P*_s_) (the right part). Positive and negative iso-surfaces are shown in red and blue (see text), respectively.

To investigate the terahertz-electric-field-induced changes of the π-electron system, we next performed terahertz-pulse-pump optical-reflectivity-probe spectroscopy in the visible region (Fig. 1c). The gray line in Fig. 3a shows the original reflectivity (*R*) spectrum with the electric fields of light *E*//*c*. As previously reported in ref.^21^, this spectrum can be reproduced by assuming two Lorentz oscillators as shown by the red broken line. The resonant energy and the dipole moment of the lower (higher)-energy transition are $\hslash {\omega }_{1}$ = 3.08 eV ($\hslash {\omega }_{2}$ = 3.41 eV) and ${\mu }_{10}=1.13\,{\rm{\AA }}$ $({\mu }_{20}=\,0.63\,{\rm{\AA }})$, respectively (see Supplementary Note 2). These two transitions are both attributed to the π-π^*^ transitions which are denoted as π-π^*^1 and π-π^*^2. Figure 3b shows the time evolutions of the terahertz-electric-field-induced reflectivity changes ${\rm{\Delta }}R(t)/R$ at 3.1 and 4.0 eV. In this experiment, the direction of ${E}_{{\rm{THz}}}(0)$ is opposite to the macroscopic polarization *P*_s_ $({E}_{{\rm{THz}}}(0)$//$-{P}_{{\rm{s}}})$. The maximum electric field |${E}_{{\rm{THz}}}(0)$| is ~210 kV/cm. Although the signs of the ${\rm{\Delta }}R(t)/R$ signals at two probe energies are reversed from each other, their time evolutions almost agree with the terahertz electric-field waveform ${E}_{{\rm{THz}}}({\rm{t}})$ or −${E}_{{\rm{THz}}}(t)$ (red lines). The probe-energy dependence of ${\rm{\Delta }}R(0)/R$ is shown by green circles in Fig. 3c. By assuming the red shifts of the π-π^*^1 and π-π^*^2 transitions by 1.3 meV, we can almost reproduce the ${\rm{\Delta }}R(0)/R$ spectrum, as shown by the red line (see Supplementary Note 2). Namely, the π-π^*^ transition energies are decreased by the electric field of ${E}_{{\rm{THz}}}(0)$//$-{P}_{{\rm{s}}}$. A previous study of SHG spectroscopy in croconic acid shows that the dipole moment of each molecule in the lowest excited state π^*^1 has the opposite direction to the original polarization *P*_s_ in the ground state^21^. In our experiment (Fig. 3) in which ${E}_{{\rm{THz}}}(0)$ is anti-parallel to *P*_s_, the energies of the ground state and the lowest excited state are expected to show higher-energy and lower-energy shifts respectively, as shown by the blue arrows in Fig. 3d. This results in the red shift of two π-π^*^ transitions as experimentally observed.

### Theoretical calculations of electric-field-induced changes in molecular orbitals

To interpret the observed electric-field-induced shifts of the π-π^*^ transitions more strictly, we theoretically investigated the electric-field dependence of molecular orbitals by means of density functional theory (DFT) calculation^22–25^ in a five-molecule cluster of H_2_C_5_O_5_ using the software package of Gaussian 09 (ref.^26^), the details of which are reported in Supplementary Note 3. In the left panel of Fig. 3d, we show the electron-density distributions (EDDs) in the highest occupied π-molecular orbital (HOMO), and the lowest and second-lowest unoccupied π-molecular orbital (π^*^1 and π^*^2) associated with the π-π^*^1 and π-π^*^2 transitions respectively. The iso-surface value of EDD is 2.70 × 10^−3^ e/Å^3^. In the HOMO, the EDD of the C=C bond near the protons is larger than that of the C-C bond on the opposite side. In the right part of Fig. 3d, we show the increase (decrease) in EDD by a static electric field *E*_s_ = 5 MV/cm, which is right-pointing in the figure and opposite to the spontaneous polarization (*E*_s_//−*P*_s_). The red and blue surfaces are the iso-surfaces with 1.35 × 10^−4^ and −1.35 × 10^−4^ e/Å^3^, respectively. In the ground state, the EDD of the C=C bond decreases and that of the C-C bond on the opposite side increases due to the electric field. This suggests that the applied electric field induces intramolecular π-electron transfer, which is responsible for the terahertz-electric-field-induced polarization changes experimentally observed. The energy shift of the π-π^*^1 transition was calculated to be ~1 meV at 210 kV/cm, which is almost equal to the experimental value (1.3 meV) (see Supplementary Note 3). This supports the validity of our interpretation.

### Theoretical calculations of IR-active vibrational modes

Another important issue is to clarify the contribution of proton dynamics to the electric-field-induced polarization changes. To do this, we measured the frequency changes of O-H and C=O stretching modes in the mid-IR region by the electric field, which are reported in the next section. To interpret those results, we need to identify the IR-active molecular vibrations associated with O-H and C=O stretching modes in the experimental IR spectrum. In this section, we report the results of the theoretical calculations of the IR-active molecular vibrations, and compare them to the experimental IR spectrum.

**Figure 4:**
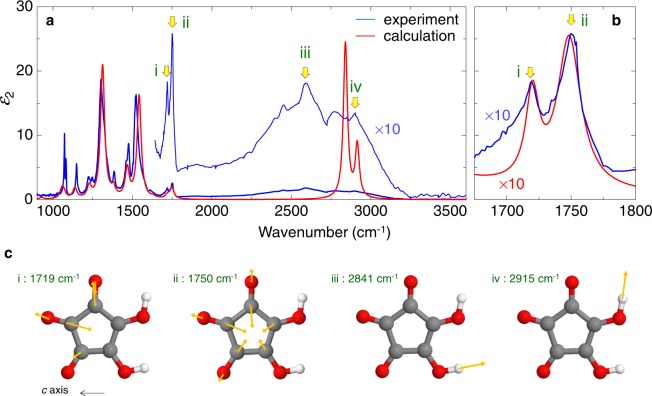
Experimental and calculated IR spectra of imaginary part of the dielectric constant ***ε***_**2**_ and calculated IR active modes. (**a**) ${\varepsilon }_{2}$ spectra in the mid-IR range. In the calculated spectrum, the spectral width is set to be 32 cm^−1^. (**b**) Expanded ${\varepsilon }_{2}$ spectra in the region of C=O stretching modes. In the calculated spectrum, the spectral width is set to be 13 and 23 cm^−1^ for (i) and (ii) bands, respectively, so as to fit the experimental spectrum. (**c**) Calculated IR-active modes associated with O-H and C=O stretching vibrations.

**Figure 5:**
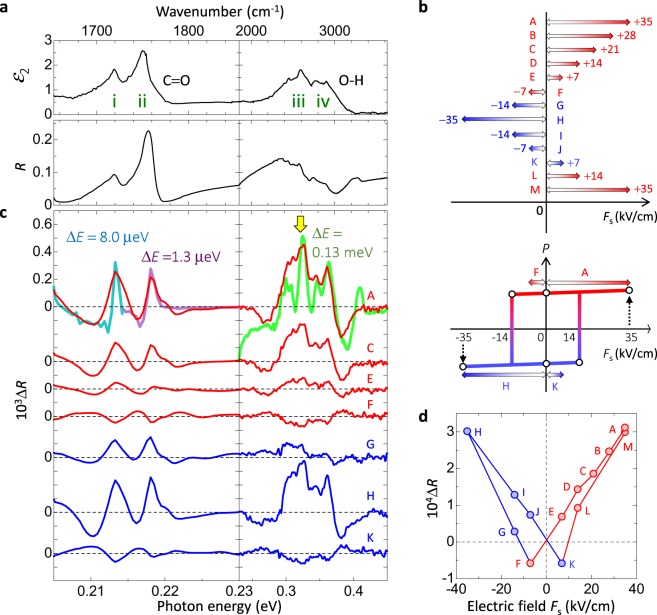
Electric-field-induced reflectivity changes in the mid-IR region. (**a**) Polarized reflectivity and ${\varepsilon }_{2}$ spectra for *E*//*c*. (**b**) The measurement sequence of the static-electric-field (*F*_s_)-induced reflectivity changes Δ*R*. The polarization versus electric-field curve in the lower part is schematic. (**c**) Δ*R* spectra for *E*//*c*. The green, purple, and sky-blue lines show −$\frac{{\rm{d}}R}{{\rm{d}}E}{\rm{\Delta }}E$ spectra (see text). (**d**) ${\rm{\Delta }}R$ values at 2600 cm^−1^ (the arrow in (**c**)) as a function of electric fields *F*_s_.

In Fig. 4a,b, we show by blue lines the spectrum of the imaginary part $({\varepsilon }_{2})$ of the dielectric constant in the mid-IR region, which is obtained by the Kramers–Kronig transformation of the polarized reflectivity spectrum for *E*//*c* in croconic acid (the lower panels of Fig. 5a). We focus on the higher-frequency bands (i)–(iv) indicated by yellow arrows in Fig. 4a,b.

For the calculations of the IR molecular vibrational modes, we used the CRYSTAL14 package^27^. Here, we adopted the same density functional and the basis set that were used in the calculations by Gaussian 09 mentioned above (see Supplementary Note 3). As the first step in the calculation, we optimized the atomic positions by fixing the cell parameters at those of the experimentally determined structure^2^. The obtained atomic positions are close to the experimental values. This fact justifies the present treatments, i.e., the selections of the density functional and the basis set, and the fixing of the cell parameters.

Next, we performed vibrational analysis and obtained the ${\varepsilon }_{2}$ spectra of the IR-active vibrations. In Fig. 4a, we show the calculated ${\varepsilon }_{2}$ spectrum by the red line, in which the spectral widths are set to be 32 cm^−1^ in all modes. For the calculated spectrum, the wavenumber is scaled by an empirical factor appropriate for B3LYP and 6–31G(d), namely, 0.96 (ref.^28^). Below 1800 cm^−1^, the calculated spectrum almost reproduces the peak positions and relative intensities of each mode in the experimental spectrum. When the spectral width of each mode is varied, the accordance of the experimental and calculation results can be improved. In Fig. 4b, we set the spectral widths of the two bands peaked at (i) 1719 and (ii) 1750 cm^−1^ to be 13 and 23 cm^−1^, respectively. In this case, the calculated spectrum better reproduces the experimental spectrum (the bands at 1720 cm^−1^ and 1748 cm^−1^) as shown by the red line. In the higher frequency region, the calculated spectrum gives two peaks at 2841 and 2915 cm^−1^, the frequencies and spectral widths of which are considerably different from those experimentally observed.

To characterize the four high-frequency modes (i)-(iv) obtained by the theoretical calculations, we show the directions and relative magnitudes of atomic displacements in each mode in Fig. 4c using orange arrows. The modes with 2841 cm^−1^ and 2915 cm^−1^ are the vibrations of the O-H group that forms the hydrogen bond along the *c* axis in the right-lower part of the molecule and along the diagonal direction in the right-higher part of the molecule, respectively (Fig. 4c). By comparing the calculated and experimental O-H stretching mode frequencies, we assigned the bands (iii) and (iv) to the dynamics of O-H groups in the right-lower part and in the right-higher part of molecule, respectively (Fig. 4c). The difference in the experimental and calculated frequencies of those two modes related to the hydrogen-bonds is attributable to the fact that the theoretical calculation is based on the harmonic approximation. In a real system, protons usually show anharmonic dynamics, which is difficult to reproduce quantitatively in the framework used in this study.

The modes with 1750 cm^−1^ and 1719 cm^−1^ consists of combinations of C=O stretching vibrations as shown in Fig. 4c. The former mode includes both of two C=O stretching vibrations that take part in the hydrogen-bonds (O−H$\cdots$O=C), while the latter includes only one such C=O stretching vibration. The frequencies of the two IR-active modes depend on how those C=O stretching vibrations are combined.

### Electro-reflectance spectroscopy to detect electric-field-induced changes in O-H bonds

For the evaluation of the frequency changes of O-H and C=O stretching modes (Fig. 4c) by the electric field, a measurement with high frequency resolution is necessary, so that we exploited Fourier Transform IR spectroscopy (FTIR) under static electric fields instead of terahertz-pulse-pump mid-IR-probe spectroscopy. Figure 5a shows the *R* spectrum (*E*//*c*) under no external electric fields (*F*_s_), and the ${\varepsilon }_{2}$ spectrum (the same as that in Fig. 4a,b). To obtain electric-field-induced reflectivity changes ${\rm{\Delta }}R$ in the mid-IR region, we measure an *R* spectrum under a static field *F*_s_ and alternately, in the absence of an electric field (see Methods). By setting *F*_s_ from A (*F*_s_ = +35 kV/cm) to H (*F*_s_ = −35 kV/cm) and then from H (*F*_s_ = −35 kV/cm) to M (*F*_s_ = +35 kV/cm) in that order as shown in Fig. 5b, we obtain the ${\rm{\Delta }}R$ spectra for each *F*_s_, a part of which is shown in Fig. 5c. The spectral shapes of ${\rm{\Delta }}R$ associated with bands (i)-(iv) at A (*F*_s_ = +35 kV/cm) in the top of Fig. 5c are almost reproduced by −$\frac{{\rm{d}}R}{{\rm{d}}E}{\rm{\Delta }}E$ with ${\rm{\Delta }}E=8.0\,{\rm{\mu }}{\rm{e}}{\rm{V}}$ for (i), ${\rm{\Delta }}E=1.3\,{\rm{\mu }}{\rm{e}}{\rm{V}}$ for (ii), ${\rm{\Delta }}E=0.13\,{\rm{meV}}$ for (iii) and (iv). These results indicate the blue shifts of both O-H and C=O stretching modes and, therefore, the decrease of the O-H and C=O bond lengths by the electric field. These phenomena can be explained as when *F*_s_ is much larger than the coercive field *F*_c_, the polarization is aligned parallel to *F*_s_ within its first application. Protons and oxygen ions move parallel and anti-parallel to *F*_s_, respectively, decreasing both the O-H and C=O bond lengths. To clearly highlight the electric-field dependence of the responses in Fig. 5c, we plot ${\rm{\Delta }}R$ values at 2600 cm^−1^ against *F*_s_ in Fig. 5d. The characteristic butterfly trace corresponds well to the hysteresis behaviour of the polarization.

## Discussion

From the experimental results of the terahertz-pulse-pump optical-reflectivity-probe spectroscopy and the theoretical calculations of electric-field-induced changes in molecular orbitals, we can conclude that *π*-electron wavefunctions are modulated by terahertz electric field. An important issue is to evaluate the magnitude of the terahertz-electric-field-induced change in the polarization of *π*-electron wavefunctions, *P*_*π*_, in the ground state. This can be deduced from the ${\rm{\Delta }}{I}_{{\rm{SHG}}}(0)/{I}_{{\rm{SHG}}}$ value. The magnitude of SHG, *I*_SHG_, is proportional to the square of the nonlinear polarization $P(\,-\,2\omega )$ with frequency $2\omega$, which is generated from the electric field of the incident light with a frequency $\omega$, $E(\omega )$, via the relation, $P(\,-\,2\omega )={\epsilon }_{0}{\chi }^{(2)}E(\omega )E(\omega )$. ${\chi }^{(2)}$ is the second-order nonlinear susceptibility and ${I}_{{\rm{SHG}}}\propto {({\chi }^{(2)})}^{2}$. In general, the SHG-intensity change ${\rm{\Delta }}{I}_{{\rm{SHG}}}(0)/{I}_{{\rm{SHG}}}$ by the terahertz electric field can be induced by the following three processes: (1) the modulation of the optical constants, that is, ${\rm{\Delta }}R(0)/R\,$ in the present case, for the fundamental probe light, (2) the modulation of the value of ${\chi }^{(2)}$, and (3) the modulation of ${\rm{\Delta }}R(0)/R$ for the SH light. The ${\rm{\Delta }}R(0)/R$ value at the photon energy (0.95 eV) of the fundamental probe light is ~0.004 at ${E}_{{\rm{THz}}}(0)$ = 150 kV/cm, which is much smaller than ${\rm{\Delta }}{I}_{{\rm{SHG}}}(0)/{I}_{{\rm{SHG}}}$ ~ 0.2 at the same electric field magnitude (not shown). As shown in Fig. 3c, the maximum value of |${\rm{\Delta }}R(0)/R$| in the range of 2.6–4.0 eV is ~0.003 at ${E}_{{\rm{THz}}}(0)$ = 210 kV/cm. Since no electronic transitions exist below 2.6 eV^21^, ${\rm{\Delta }}R(0)/R$ for the SH light at 1.9 eV and at ${E}_{{\rm{THz}}}(0)$ = 150 kV/cm would be smaller than 0.003. Therefore, we can consider that the processes (1) and (3) are not the main origin for the SHG-intensity change ${\rm{\Delta }}{I}_{{\rm{SHG}}}(0)$/${I}_{{\rm{SHG}}}$, and that the process (2) dominates that. When we assume that ${\chi }^{(2)}$ is proportional to *P*_*π*_, we obtain ${\rm{\Delta }}{I}_{{\rm{SHG}}}(0)/{I}_{{\rm{SHG}}}=2{\rm{\Delta }}{P}_{\pi }/{P}_{\pi }$. Using this formula and the experimental result $(|{\rm{\Delta }}{I}_{{\rm{SHG}}}(0)/{I}_{{\rm{SHG}}}|\sim 20 \% )$, we evaluate the terahertz-electric-field-induced change of *P*_*π*_ at the time origin, ${\rm{\Delta }}{P}_{\pi }(0)$/${P}_{\pi }$, to be ~10% at ${E}_{{\rm{THz}}}(0)=150$ kV/cm. The details of the analyses are reported in Supplementary Note 4.

On the other hand, we can evaluate the change ${\rm{\Delta }}{\delta }_{{\rm{H}}}$ in the displacement *δ*_H_ of protons that form the hydrogen-bonds. Here, *δ*_H_ is simply defined by $(d({\rm{O}}\cdots {\rm{H}})-d({\rm{O}}-{\rm{H}}))$/2. From the magnitudes of the blue shift ${\rm{\Delta }}\hslash {\omega }_{{\rm{OH}}}$ of the O-H stretching-mode frequency $\hslash {\omega }_{{\rm{OH}}}$, and the empirical relation between $\hslash {\omega }_{{\rm{OH}}}$ and *δ*_H_, ${\rm{\Delta }}{\delta }_{{\rm{H}}}$ is estimated to be ~$1.6\times {10}^{-4}\,{\rm{\AA }}$ at ${F}_{{\rm{s}}}=+\,35$ kV/cm. The details of the analysis are reported in Supplementary Note 5. ${\rm{\Delta }}{\delta }_{{\rm{H}}}$ is negligibly small compared to the original value, ${\delta }_{{\rm{H}}}\,(\,\sim \,0.31\,{\rm{\AA }})$, which suggest that proton displacements do not contribute to the electric-field-induced polarization changes. It is probably because each proton position is determined by the positions of oxygen atoms that form the $({\rm{O}}-{\rm{H}}\cdots {\rm{O}})$ hydrogen-bond, which are hardly changed when each molecular position is fixed.

These results contradict the simple prediction that protons collectively move along with π-electrons when external electric fields are applied. Instead, they demonstrate that the large electric-field-induced polarization modulation observed is caused only by the π-electron-wavefunction changes, which are characterized by ${\rm{\Delta }}{P}_{\pi }(0)$/${P}_{\pi }$. The results also suggest that the ferroelectric polarization of croconic acid in the steady-state can also be ascribed to the π-electron polarizations. This is consistent with a recent theoretical study using first-principle calculation, which suggests that *π*-electron polarization reaches 80% of *P*_s_ (ref.^6^). As a result, ferroelectricity of croconic acid can be efficiently controlled in the sub-picosecond time scale by a terahertz electric field. If the amplitude of the terahertz pulse is enhanced up to ~2 MV/cm, the polarization modulation with ${\rm{\Delta }}{P}_{\pi }(0)$/${P}_{\pi }\sim 1$ might be achieved. The enhancement of the electric-field amplitude of the pulse is possible in the mid-infrared region^29^. The polarization control using such a strong mid-infrared pulse is an interesting subject in future.

In summary, in an organic molecular ferroelectric of croconic acid, it was demonstrated that a large polarization modulation in the sub-picosecond time scale is possible by using a terahertz electric-field pulse. The modulation amplitude reached 10% of the original ferroelectric polarization, which is ascribed to the field-induced modifications of π-electron wavefunctions. The DFT calculations of π-electron wavefunctions under electric fields support this interpretation. The contribution of proton displacements in the hydrogen-bonds is negligibly small. Such an ultrafast polarization control via π-electron systems is expected to be also possible in many other hydrogen-bonded molecular ferroelectrics and can be utilized for future high-speed optical-modulation devices.

## Methods

### Sample preparation

Single crystals of croconic acid were synthesized by a previously reported method^2^. A typical size of the samples used is ~400 μm × 400 μm (*ac* plane) and the thickness is ~200 μm.

### Pump probe measurements

In the terahertz-pulse-pump SHG-probe and optical-reflectivity-probe measurements, a Ti:sapphire regenerative amplifier (RA) with a pulse width of 130 fs, wavelength of 800 nm, and pulse energy of 2 mJ was used as a light source. The output of the RA was divided into three beams. The first beam was used as an excitation source of a terahertz radiation system in which a strong terahertz pulse is generated *via* optical rectification in a LiNbO_3_ crystal by the pulse-front tilting method^30,31^. The second beam was used for the electro-optical sampling to detect the electric-field waveform of the terahertz pulse. The generation and detection methods of terahertz pulses are reported elsewhere^32^. The third beam was used to excite an optical parametric amplifier, from which we obtained probe pulses of 0.95 eV for the SHG-probe measurement and of 2.6–4.0 eV for the optical-reflectivity-probe measurement. The delay time *t* of the probe pulse relative to the terahertz pump pulse was controlled by changing the path length of the probe pulse. The time origin (*t* = 0) was defined as the time when the terahertz electric field reached a maximum value. Pump and probe pulses were incident to the *ac* plane of a crystal. All the experiments were performed in a vacuum at 294 K.

### Electro-reflectance spectroscopy

We measured mid-IR reflectivity changes ${\rm{\Delta }}R$/$R$ under static electric fields condition using an FTIR spectrometer equipped with an optical microscope^33^. Two electrodes with carbon pastes were placed on both sides of a crystal (*ab* planes) (Fig. 1d) and a static electric field was applied along the *c* axis. The *R* spectrum was first measured for 30 sec under an applied electric field *F*_s_ and then with no electric field for 30 sec. To improve the signal to noise ratio, this cycle was repeated for several hundred times.

## Electronic supplementary material

Supplementary Information

## Data Availability

The data that support the findings of this study are available from the corresponding author on request.
