# Supplementary material for: Ultrafast polarization control by terahertz fields via π-electron wavefunction changes in hydrogen-bonded molecular ferroelectrics
Source: Sci Rep. 2018 Oct 9;8:15014. doi: 10.1038/s41598-018-33076-9 (PMC6177455; doi:10.1038/s41598-018-33076-9)
Supplement: Supplementary file 1 — Supplementary Information [file 41598_2018_33076_MOESM1_ESM.pdf]

# Supplementary Information:

# Ultrafast polarization control by terahertz fields via π-electron wavefunction changes in hydrogen-bonded molecular ferroelectrics

T. Miyamoto[1], D. Hata[1], T. Morimoto[1], H. Yamakawa[1], N. Kida[1], T. Terashige[2], K. Iwano[3], H. Kishida[4], S. Horiuchi[5] & H. Okamoto[1,2]

[1]Department of Advanced Materials Science, University of Tokyo, Kashiwa, 277-8561, Japan

[2]AIST-UTokyo Advanced Operando-Measurement Technology Open Innovation Laboratory (OPERANDO-OIL), National Institute of Advanced Industrial Science and Technology (AIST), Chiba 277-8568, Japan

[3]Graduate University for Advanced Studies, Institute of Materials Structure Science High Energy Accelerator Research Organization (KEK), Tsukuba 305-0801, Japan

[4]Department of Applied Physics, Nagoya University, Nagoya 464-8603, Japan

[5]National Institute of Advanced Industrial Science and Technology (AIST), Tsukuba 305-8562, Japan

**Contents**

**S1. Terahertz electric-field dependence of SHG-intensity changes**

**S2. Simulations of reflectivity spectrum and reflectivity changes by terahertz electric fields**

**S3. Theoretical calculations of molecular orbitals and π-π* transitions under electric fields**

**S4. Analyses of terahertz-electric-field-induced SHG changes**

**S5. Evaluations of electric-field-induced proton displacements from IR spectroscopy**

## S1. Terahertz electric-field dependence of SHG-intensity changes

We measured the changes of SHG intensities $\Delta I_{\mathrm{SHG}}(t)/I_{\mathrm{SHG}}$ for various amplitudes of terahertz electric fields, $E_{\mathrm{THz}}(0)$. Figure S1 shows the $E_{\mathrm{THz}}(0)$-dependence of $\Delta I_{\mathrm{SHG}}(0)/I_{\mathrm{SHG}}$, which is proportional to $E_{\mathrm{THz}}(0)$ up to 240 kV/cm.

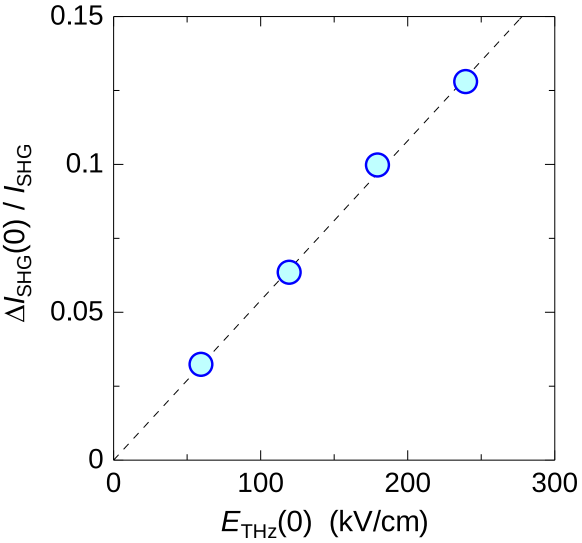


**Figure S1.** Terahertz-electric-field $E_{\mathrm{THz}}(0)$ dependence of SHG intensity changes $\Delta I_{\mathrm{SHG}}(0)/I_{\mathrm{SHG}}$.

## S2. Simulations of reflectivity spectrum and reflectivity changes by terahertz electric fields

A previous study reported that the polarized reflectivity$(R)$ spectrum along the *c* axis consists of two Lorentz oscillators[1]. We performed the fitting analysis using the dielectric function,

$$\varepsilon(\omega) = \varepsilon_{\infty} + \frac{Ne^2}{\hbar} \sum_{i=1,2} \langle 0|x|i\rangle^2 \left( \frac{1}{\omega_i - \omega - i\gamma_i} + \frac{1}{\omega_i + \omega + i\gamma_i} \right). \qquad \text{(S1)}$$

Here, $|0\rangle$, $|1\rangle$, and $|2\rangle$ are the wavefunctions of the ground state, the lowest excited state π*1, and the second-lowest excited state π*2, respectively. $\hbar\omega_i$ is the resonant

energy of the π-π*$i$ transition and $\hbar\gamma_i$ is the damping constant of state $|i\rangle$. $\langle i|x|0\rangle (= \mu_{i0})$ is the transition dipole moment between $|0\rangle$ and $|i\rangle$. The calculated reflectivity spectrum is shown by the red broken line in Fig. 3a, which reproduces well the experimental $R$ spectrum (the gray line). The obtained parameter values are listed in Table S1, which are almost equal to those reported in Ref [1]. $\langle 2|x|0\rangle (= 0.63 \text{ Å})$ is considerably smaller than $\langle 1|x|0\rangle (= 1.13 \text{ Å})$.

To explain the observed terahertz-electric-field-induced reflectivity changes $\Delta R(0)/R$ at the time origin $(E_{\text{THz}}(0) \sim 210 \text{ kV/cm})$ shown by the green circles in Fig. 3c, we assume that the transition energies $\hbar\omega_1$ and $\hbar\omega_2$ decrease due to the terahertz electric field (see the main text). Note that, in our case, the electric-field direction at the time origin is opposite to the ferroelectric-polarization direction $(E_{\text{THz}}(0)//-P_{\text{s}})$, as indicated in the lower part in Fig. 3d. We decrease $\hbar\omega_1$ and $\hbar\omega_2$ by 1.3 meV from the original values and calculate the change of the reflectivity spectrum $\Delta R(0)/R$ by using the dielectric function (equation (S1)) with the other parameter values fixed. The result is shown by the red line in Fig. 3c. This spectrum almost reproduces the $\Delta R(0)/R$ spectrum experimentally obtained.

**Table S1.** Parameter values evaluated from the analyses of the reflectivity spectrum along the *c* axis.

| $\varepsilon_\infty$ | $\langle 0\|x\|1\rangle$ | $\langle 0\|x\|2\rangle$ | $\hbar\omega_1$ | $\hbar\omega_2$ | $\hbar\gamma_1$ | $\hbar\gamma_2$ |
|---|---|---|---|---|---|---|
| 2.07 | 1.13 Å | 0.63 Å | 3.08 eV | 3.41 eV | 0.162 eV | 0.311 eV |

## S3. Theoretical calculations of molecular orbitals and π-π* transitions under electric fields

In this section, we show the details of the theoretical calculations of the π-molecular orbitals and π-π* transitions without and with external electric fields. We performed a density functional theory (DFT) calculation in a five-molecule cluster of $H_2C_5O_5$. Here, we used the software package of Gaussian 09 with the combination of B3LYP as the density functional and 6-31G(d) as the basis set[2]. Regarding the electronic excited states, we applied the method of time-dependent DFT[3] and analysed the behaviour of two low-lying electronic transitions at 3.08 eV and 3.41 eV. The results demonstrated that they are π-π* transitions and their energies are 3.63 eV and 3.96 eV. The differences between the experimental and calculated transition energies are attributable to finite size effects. The related molecular orbitals, the occupied π orbital, and the unoccupied π*1 and π*2 orbitals, are depicted in Fig. 3d.

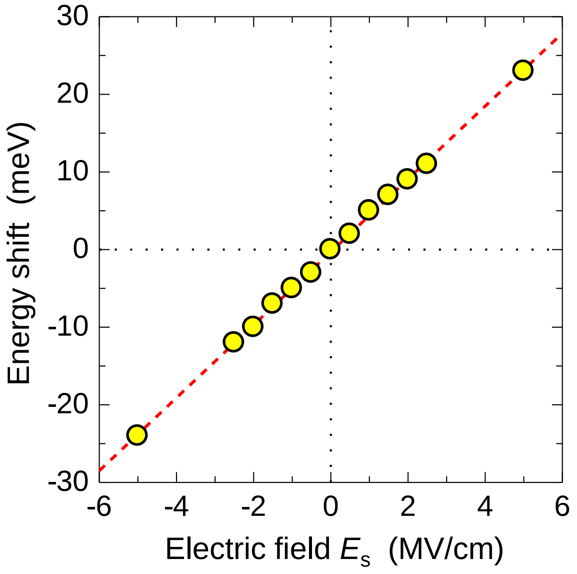


**Figure S2.** Calculated energy shifts of the π-π*1 transition as a function of electric fields $E_s$ along the $c$ axis.

In addition, we calculated molecular orbitals under static electric fields $E_{\mathrm{s}}$. In this calculation, all the atoms are fixed in position under no electric fields. Figure S2 shows the magnitudes of the energy shifts of the π-π*1 transition as a function of electric fields $E_{\mathrm{s}}$ along the *c* axis, in which electric fields parallel to the spontaneous polarization $P_{\mathrm{s}}$ is defined to be positive. The result clearly shows that the energy shift of the π-π*1 transition is proportional to $E_{\mathrm{s}}$ and is equal to ~1 meV at 210 kV/cm. This value is comparable to the experimental result.

**S4. Analyses of terahertz-electric-field-induced SHG changes**

The magnitude of $I_{\mathrm{SHG}}$ is dominated by the second-order nonlinear susceptibility $\chi^{(2)}(-2\omega;\omega,\omega)$, which is simply denoted as $\chi^{(2)}$ in the main text. Using a simple two-level model[4], $\chi^{(2)}(-2\omega;\omega,\omega)$ in croconic acid is expressed by the wavefunctions of the ground state $(|0\rangle)$, and of two excited states π*1 $(|1\rangle)$ and π*2 $(|2\rangle)$ as follows [30].

$$\chi^{(2)}(-2\omega;\omega,\omega) \propto \sum_{i=1,2} \Delta p_{i0} \times f_i(\omega),$$

$$f_i(\omega) = |\langle i|x|0\rangle|^2 \times$$

$$\left\{\frac{[2\omega^2+\omega_i{}^2-(\gamma_i/2)^2]+3i\omega\gamma_i}{[(2\omega+i\gamma_i)^2-\omega_i{}^2][(\omega+i\gamma_i)^2-\omega_i{}^2]}-\frac{2(2\omega^2+2i\omega\gamma_i)}{(2\omega)^2[(\omega+i\gamma_i)^2-\omega_i{}^2]}\right\} \qquad (S2)$$

Here, $\Delta p_{i0} (= \mu_i - \mu_0)$ is the difference between the dipole moments of the ground state $\mu_0 = \langle 0|x|0\rangle$ and the excited state $\mu_i = \langle i|x|i\rangle$. From the parameter values in Table S1, $f_2(\omega)$ is calculated to be one-sixth of $f_1(\omega)$. This indicates that the nonlinear optical response is dominated by the π-π*1 transition and $f_2(\omega)$ (the contribution of the π-π*2 transition) can be neglected. Here, it is natural to consider that $\mu_0$ is proportional to $P_\pi$,

since $\mu_0$ is the electronic dipole moment related with π-electron wavefunctions in the ground state. We also assume that $\mu_1$ is proportional to $-\mu_0$, which leads to the relation $\Delta p_{10} \propto P_\pi$. Using the relations $\chi^{(2)} \propto \Delta p_{10}$ and $I_{\mathrm{SHG}} \propto \left(\chi^{(2)}\right)^2$, we obtain $I_{\mathrm{SHG}} \propto {P_\pi}^2$, which leads to $\Delta I_{\mathrm{SHG}}(0)/I_{\mathrm{SHG}} = 2\,\Delta P_\pi/P_\pi$. From this relation and the experimental result $(|\Delta I_{\mathrm{SHG}}(0)/I_{\mathrm{SHG}}|{\sim}20\%)$, we obtain $\Delta P_\pi(0)/P_\pi{\sim}10$ % at $E_{\mathrm{THz}}(0) = 150$ kV/cm, as reported in the main text.

In this evaluation, we neglect electric-field-induced changes of the other parameters such as $\hbar\omega_1$ and $\mu_{10} = \langle 1|x|0\rangle$, which may be changed. As discussed in the main text and the previous section S2, we can reproduce the terahertz-electric-field-induced spectral change of the reflectivity $\Delta R(0)/R$ only by decreasing $\hbar\omega_1$ and $\hbar\omega_2$ as shown by the red line in Fig. 3c. This suggests that the change of the spectral weight itself is small and therefore we can consider that $\langle 1|x|0\rangle$ is almost unchanged by the terahertz electric field. The shift of $\hbar\omega_1$ (1.3 meV at $E_{\mathrm{THz}}(0){\sim}210$ kV/cm) is not the main origin of the change in $\chi^{(2)}(-2\omega;\omega,\omega)$, since $\Delta\chi^{(2)}/\chi^{(2)}$ and $\Delta I_{\mathrm{SHG}}/I_{\mathrm{SHG}}$ by this effect are estimated to be $\sim 1.4 \times 10^{-3}$ and $\sim 2.8 \times 10^{-3}$, respectively, from $\Delta p_{10} = 5.3$ Å (Ref [1]), $\hbar\omega_1 = 3.08$ eV, $\hbar\gamma_1 = 0.16$ eV and $\Delta\hbar\omega_1 = 1.3$ meV. Thus, we conclude that the change of $\Delta p_{10}$ is the main origin of the terahertz-electric-field-induced change of $\chi^{(2)}$ and $\Delta I_{\mathrm{SHG}}(0)/I_{\mathrm{SHG}}$.

**S5. Evaluations of electric-field-induced proton displacements from IR spectroscopy**

In this section, we detail the evaluation of proton displacement induced by the electric field from the frequency shift of the O-H stretching vibrations. For this analysis, we first

review the relation between the O-H bond length, $d(\mathrm{O-H})$, and the frequency, $\nu(\mathrm{O-H})$, of O-H stretching vibrations, which was previously reported.

In molecular crystals with intermolecular hydrogen-bonds, the following empirical relation (relation I) exist between $d(\mathrm{O-H})$ and the O···H distance, $d(\mathrm{O\cdots H})$.

$$d(\mathrm{O\cdots H}) = r_0 - c \times \log_{10}\left(1 - 10^{-(d(\mathrm{O-H})-r_0)/c}\right)\ [\text{Å}]$$

Here, $r_0 = 0.925$ and $c = 0.9135$ (Ref [5]). This formula shows the tendency for $d(\mathrm{O-H})$ to increases with a decrease of $d(\mathrm{O\cdots H})$. In addition, $d(\mathrm{O-H})$ can be expressed in terms of Pauling's bond order[6] $n(\mathrm{O-H})$ by the following formula (relation II)[5].

$$d(\mathrm{O-H}) = r_0 - c \times \log_{10} n(\mathrm{O-H})\ [\text{Å}]$$

It is also known that a linear relation (relation III) exist between $\nu(\mathrm{O-H})$ and $n(\mathrm{O-H})$ (Ref [5]).

$$\nu(\mathrm{O-H}) = -6140 + 10700 \times n(\mathrm{O-H})\ [\mathrm{cm^{-1}}]$$

Using relations II and III, we obtain a relation between $d(\mathrm{O-H})$ and $\nu(\mathrm{O-H})$.

Here, we focus on the hydrogen-bond along the *c* axis in croconic acid and the corresponding O-H stretching band (iii) (see Figs. 4 and 5). This is because both the spontaneous polarization and the applied electric field are parallel to the *c* axis and the electric-field-induced change of the reflectivity is measured for $E\ //\ c$. In this hydrogen-bond along the *c* axis, $d(\mathrm{O-H})$ and $d(\mathrm{O\cdots H})$ are evaluated to be 1.0 Å, and 1.6 Å, respectively, from the X-ray and neutron diffraction studies[7]. By substituting $d(\mathrm{O-H}) = 1.0$ Å into relation I, we obtain $d(\mathrm{O\cdots H}) = 1.62$ Å, which is almost equal to the experimental value. Using relations II and III, and $d(\mathrm{O-H}) = 1.0$ Å, we obtain

$\nu(\mathrm{O-H}) = 2717\ \mathrm{cm}^{-1}$, which is close to the O-H stretching mode frequency of band (iii).

As reported in the main text, the shift of the O-H stretching-mode frequency $\nu(\mathrm{O-H})$, $\Delta\nu(\mathrm{O-H})$, by changing the static electric field $F_s$ from 0 to 35 kV/cm was 0.13 meV (1.0 $\mathrm{cm}^{-1}$). Using this value and the relations II and III, the electric-field-induced changes of $\delta_\mathrm{H}$, $\Delta\delta_\mathrm{H}$, is evaluated to be ~$1.6\times10^{-4}$ Å. Using $\delta_\mathrm{H} = 0.31$ Å we obtain $\Delta\delta_\mathrm{H}/\delta_\mathrm{H} = 5.0\times10^{-4}$ at $F_s = 35$ kV/cm. As reported in the main text, the polarization change that originates from the change of the π-electron wavefunctions, $\Delta P_\pi/P_\pi$, is ~0.10 at $E_\mathrm{THz}(0)$~150 kV/cm. Assuming a linear relation between $\Delta P_\pi/P_\pi$ and $E_\mathrm{THz}(0)$, $\Delta P_\pi/P_\pi$ at $E_\mathrm{THz}(0) = 35\ \mathrm{kV/cm}$ is $2.3\times10^{-2}$. The obtained $\Delta\delta_\mathrm{H}/\delta_\mathrm{H}$ is more than one order of magnitude smaller than $\Delta P_\pi/P_\pi$. The parameter values of $d(\mathrm{O-H})$, $d(\mathrm{O\cdots H})$, $\Delta\delta_\mathrm{H}$ $\Delta\delta_\mathrm{H}/\delta_\mathrm{H}$ and $\Delta P_\pi/P_\pi$ at $F_s = 35$ kV/cm are listed in Table S2.

**Table S2.** Parameter values of the O-H bond lengths, $d(\mathrm{O-H})$ and $d(\mathrm{O\cdots H})$, and proton displacement $\delta_\mathrm{H}$ in the hydrogen bonds along the *c* axis. $\Delta\delta_\mathrm{H}/\delta_\mathrm{H}$ and $\Delta P_\pi/P_\pi$ are the changes of proton displacements and π-electron polarization for the external electric field of 35 kV/cm.

| $d(\mathrm{O-H})$ | $d(\mathrm{O\cdots H})$ | $\delta_\mathrm{H}$ | $\Delta\delta_\mathrm{H}$ | $\Delta\delta_\mathrm{H}/\delta_\mathrm{H}$ | $\Delta P_\pi/P_\pi$ |
|---|---|---|---|---|---|
| 1.0 Å | 1.6 Å | 0.31 Å | $1.6\times10^{-4}$ Å | $5.0\times10^{-4}$ | $2.3\times10^{-2}$ |

**References**


1. Sawada, R. et al. Large second-order optical nonlinearity in a ferroelectric molecular crystal of croconic acid with strong intermolecular hydrogen bonds. *Appl. Phys. Lett.* **102,** 162901 (2013).

2. Frisch M. J. et al. Gaussian 09, Revision B.01. Gaussian Inc., Wallingford CT (2009).

3. Stratmann, R. E., Scuseria, G. E. & Frisch, M. J. An efficient implementation of time-dependent density-functional theory for the calculation of excitation energies of large molecules. *J. Chem. Phys*. **109,** 8218-8224 (1998).

4. Ouder, J. L. & Chemla, D. S. Hyperpolarizabilities of the nitroanilines and their relations to the excited state dipole moment. *J. Chem. Phys.* **66,** 2664-2668 (1977).

5. Bertolasi, V., Gilli, P., Ferretti, V. & Gilli, G. Resonance-Assisted O-H···O Hydrogen Bonding: Its Role in the Crystalline Self-Recognition of *β*-Diketone Enols and its Structural and IR Characterization. *Chem. Eur. J.* **2,** 925-934 (1996).

6. Pauling, L. Atomic Radii and Interatomic Distances in Metals. *J. Am. Chem. Soc.* **69,** 542-553 (1947).

7. Horiuchi, S. et al. Above-room-temperature ferroelectricity in a single-component molecular crystal. *Nature* **463,** 789-792 (2010).
